# Methylene Blue Spray as a Tool for Safe Thyroidectomy

**DOI:** 10.7759/cureus.73790

**Published:** 2024-11-15

**Authors:** Narendra Ballal, Manjunath S Kotennavar, Aravind V Patil, Benakatti Rajendra, Pradeep Jaju, Manjunath S Savant, Sanjeev S Rathod, Veena Ghanteppagol, Saket Shetty, Eswar Medikonda

**Affiliations:** 1 General Surgery, BLDE (Deemed to be University) Shri BM Patil Medical College Hospital and Research Centre, Vijayapura, IND; 2 General Surgery, S. Nijalingappa Medical College, Bagalkote, IND

**Keywords:** identification, methylene blue spray, parathyroid, recurrent laryngeal nerve, thyroid

## Abstract

Background

The complex surgical anatomy and intricate structural arrangement of the thyroid region pose significant challenges for surgeons in identifying the parathyroids and recurrent laryngeal nerve (RLN) during thyroid surgeries. Therefore, it is crucial to develop techniques that enhance the identification of these structures and reduce complications during thyroidectomies.

Objective

This study intends to assess the efficacy and diagnostic value of Methylene Blue dye and its usefulness in identifying, conserving and minimizing injury to parathyroid glands and recurrent laryngeal nerve during thyroidectomies.

Methods

Over two years, 66 patients had near-total, subtotal, or total thyroidectomies at the Shri BM Patil Medical College, Hospital & Research Centre, Vijayapura, India, as part of this interventional study. The time it took for various tissues to return to their natural colour after applying methylene blue dye was the principle used for safe thyroidectomy. Preoperative serum calcium and parathyroid hormone (PTH) concentrations, preoperative diagnoses, and demographic information were gathered for the study. Documentation was also kept of the histological confirmation, hospital stay following surgery, complications following surgery, variations in blood calcium and PTH concentrations on the fifth postoperative day, and any allergic reactions to methylene blue. This allowed for the calculation of equal sensitivity and specificity with negative and positive predictive values.

Results

Sixty-three (95%) of the 66 patients were female, most in their 40s-60s. Before surgery, patients’ serum PTH and calcium levels were normal, with no patients having hoarseness of voice or hypocalcemia symptoms. Postoperative hospital stays typically lasted three to five days. Two patients experienced vocal cord paresis following surgery, and one patient experienced delayed wound healing. They were eventually able to recover fully. On day five following surgery, there was no drop in serum PTH or calcium levels and no allergic reaction to methylene blue. Methylene blue showed a sensitivity of 98.46%, specificity of 97.01%, positive predictive value of 96.97%, negative predictive value of 98.48%, and overall accuracy of 97.73% when used for intraoperative structure detection.

Conclusion

Using methylene blue dye for the intraoperative identification and preservation of parathyroid glands and the recurrent laryngeal nerve is a reliable, affordable, and accessible method with good sensitivity and specificity. It makes thyroidectomy dissections less taxing and reduces the risk of complications following thyroid operations.

## Introduction

In the last decade, the magnitude of thyroid cancers has shown a dramatic hike by 62% and 48% among males and females, respectively [1]. The drift is diverse and focuses on the potential increment in several thyroidectomy surgeries in the near future. Specialists should anticipate performing surgery on vast numbers of patients in the future, utilizing productive surgical methods that result in ideal recuperation and negligible complications.

Thyroidectomy surgery is the principal treatment for thyroid disorders and malignancies. Thyroid gland illnesses are the second most frequent endocrine diseases after diabetes mellitus [2-4]. Due to complex surgical anatomy, it is an uphill task for the surgeon to detect parathyroids and recurrent laryngeal nerves (RLN). Erroneous identification of these can result in parathyroid hypofunction and damage to the RLN. Following a thyroidectomy, the incidence of temporary hypocalcemia is 36%, whereas the incidence of persistent hypocalcemia is 15% [5]. Severe difficulties with respiration, mental illnesses, and phonation result from vocal cord paralysis.

Complications following surgery increase hospital stays and patient costs. Identifying parathyroids and RLN is a laborious difficulty for the surgeon due to complex surgical anatomy and confounding structural fabrication. Using anatomical landmarks, partial gland biopsy for pathological examination, optical coherence tomography during surgery, parathyroid-specific luminescence, fine needle aspiration for parathyroid analysis, blood parathyroid hormone level measurement, and intraoperative nerve monitoring are all essential steps in protecting parathyroid and RLN during surgery [6]. The current guideline to protect the parathyroid and RLN during surgery is anatomical localization of the parathyroid. The operation procedure represents the main factor that changes the outcome. This study examines the effectiveness of topical methylene blue dye administration in the surgical field to identify the parathyroid gland and RLN during thyroidectomy precisely.

## Materials and methods

Source of data

It is an interventional study conducted from September 2022 to September 2024 for a period of two years, which included all patients admitted to the Department of Surgery at BLDE (Deemed to be University) Shri BM Patil Medical College Hospital and Research Centre, Vijayapur, India, between September 2022 and September 2024 and underwent total/sub-total/near total thyroidectomies for different thyroid disorders.

Sampling

Given that thyroid cancer is prevalent at 56% and that methylene blue spray is expected to have a sensitivity and specificity of 92.31% and 57%, respectively, to detect the parathyroid gland [5], 66 samples were needed to achieve a precision of 12% and 95% confidence.

The formula used is the following:

N= Z^2 ^p (1-p)/ Delta^2^

N will be (a+c) if we use sensitivity as p

N= (a+c)/Prevalence

Statistical analysis

The data obtained were entered into a Microsoft Excel (Microsoft® Corp., Redmond, WA) sheet, and statistical analysis was performed using Statistical Product and Service Solutions (SPSS, version 20; IBM SPSS Statistics for Windows, Armonk, NY). Results were presented as mean (median) ±SD, counts, percentages, and diagrams. Categorical variables were compared. Sensitivity, specificity, positive predictive value (PPV), negative predictive value (NPV), and accuracy were used to find the efficacy and diagnostic value. p<0.05 was considered statistically significant.

Methods

**Figure 1:**
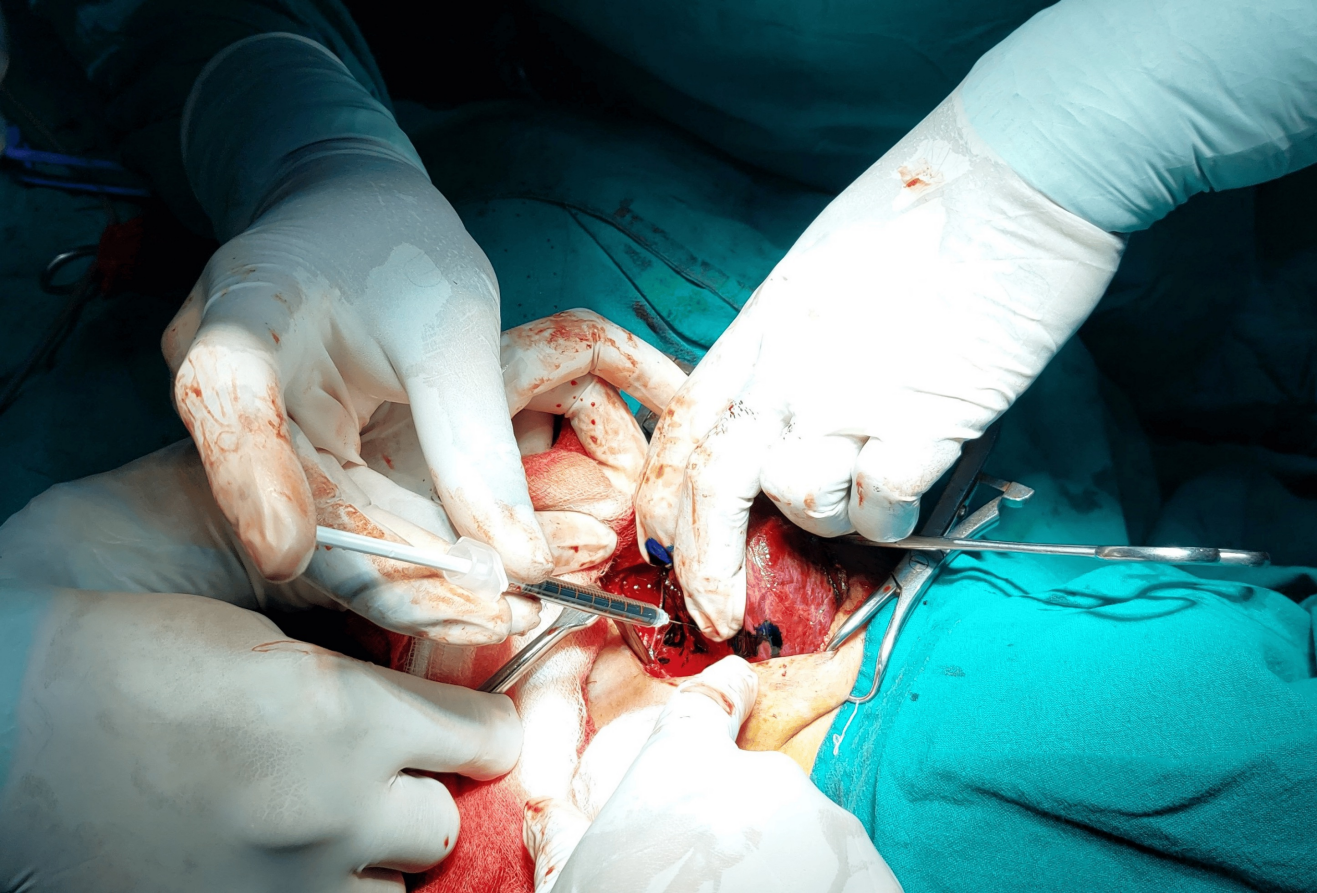
Spraying methylene blue over the thyroid bed

**Figure 2:**
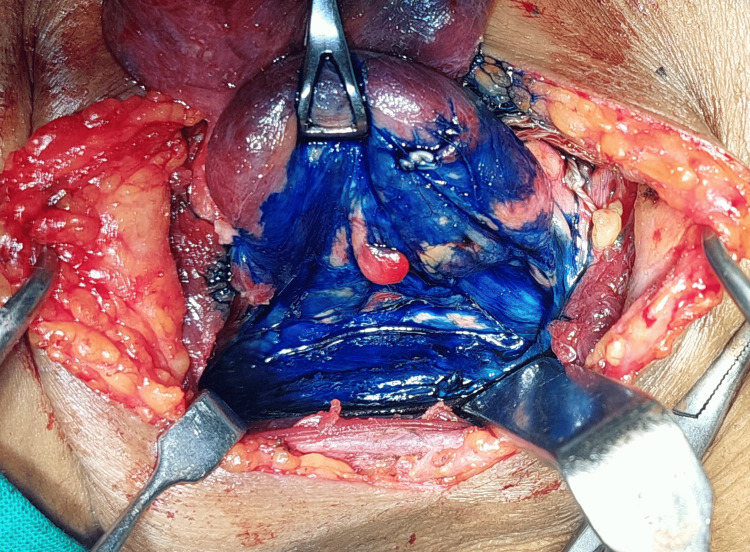
Just after spraying methylene blue

**Figure 3:**
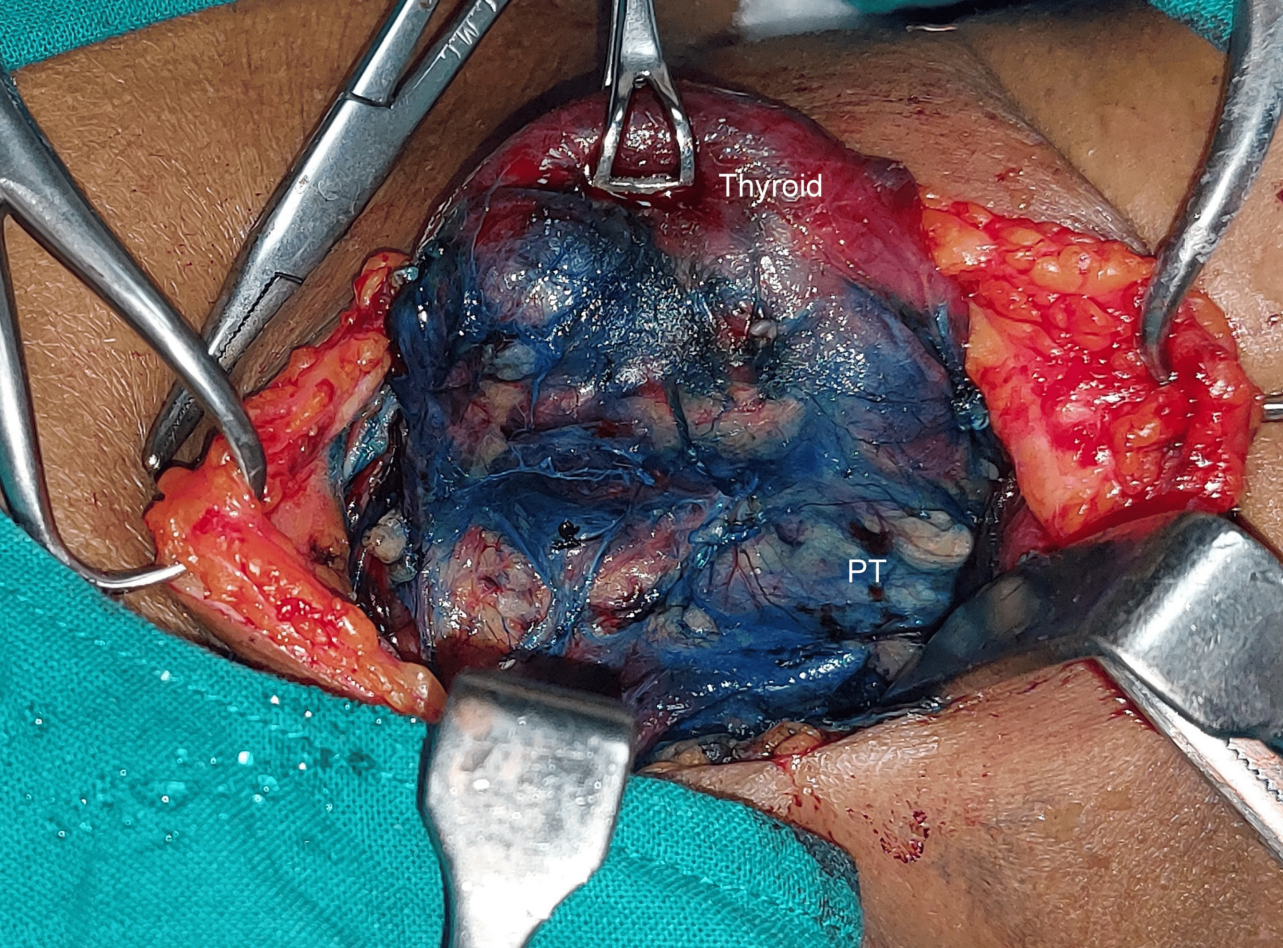
Five minutes after spraying methylene blue

**Figure 4:**
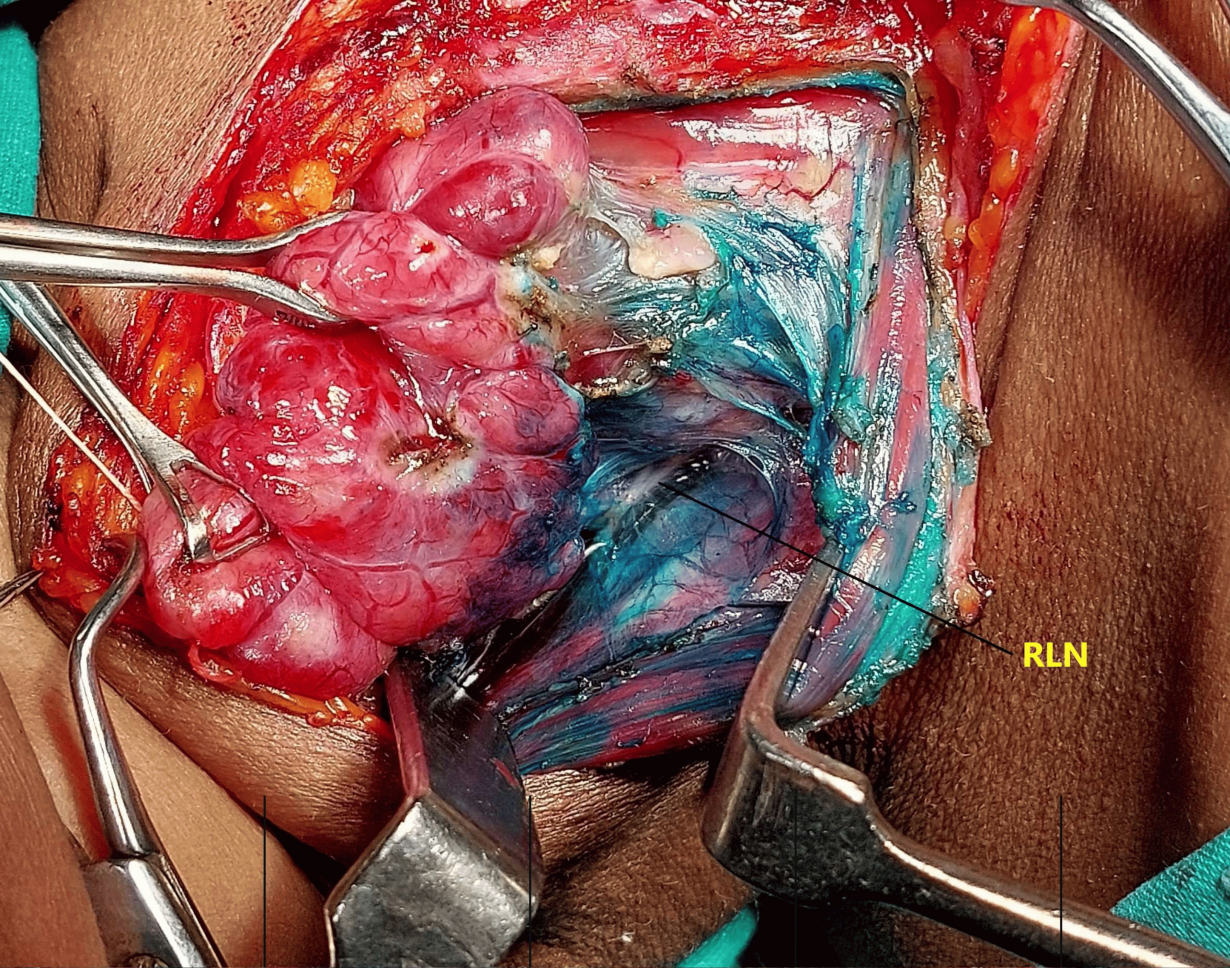
Visualization of the recurrent laryngeal nerve after spray (no dye uptake)

The formula for methylene blue is C16H18ClN3S, which is an organic chloride salt. Swiss blue and methylthioninium chloride are the other names for it. Following the exposure of the thyroid gland and the ligation of the superior pedicle and middle thyroid vein, 0.5 mL (5 mg) of 1% methylene blue solution mixed with 0.5 mL of saline was sprayed over the thyroid bed and surrounding tissue (Figures 1-2). In three to five minutes, the parathyroid gland returns to its natural yellow hue after absorbing the blue dye (Figure 3). Since the recurrent laryngeal nerve has an avascular structure covered in a Schwann sheath, it will stay unstained like any other nerve (Figure 4). It takes around 15 minutes for the thyroid, and over 25 minutes for fat, tendon, and muscle to return to their original colour. The parathyroid glands’ extensive lymphovascular architecture allows them to absorb methylene blue more quickly than other tissues.

Inclusion criteria

Cases with benign and malignant thyroid swelling admitted to BLDE (Deemed to be University) Shri BM Patil Medical College Hospital and Research Centre undergoing total/sub-total/near total/thyroidectomies were included.

Exclusion criteria

Recurrent thyroid surgery, preoperative vocal cord paresis/paralysis, retrosternal goiters, and hemithyroidectomies were excluded.

Method of collection of data

For each patient, data were collected using a pretested structured proforma. A comprehensive history was obtained from every patient. Relevant investigations included USG neck, fine needle aspiration cytology, T3, T4, TSH, serum calcium levels, and indirect laryngoscopy. Additional investigations were performed based on the patient’s history and presenting complaints. All patients provided written informed consent after a detailed explanation of the surgical procedure, including potential risks, complications, benefits, and drawbacks. Cases that met the inclusion and exclusion criteria were selected for the study. During thyroidectomy, methylene blue was used to identify the parathyroids and the recurrent laryngeal nerve (RLN). Additionally, 2 mm biopsies were performed at the region thought to represent the parathyroid gland that has returned to its original yellow hue, and the area previously supposed to symbolize the parathyroid gland has returned to being yellow. It was also confirmed by histopathology of the thyroid specimen. The study will evaluate methylene blue spray’s safety, sensitivity, specificity, and post-operative complications. Post-operative assessments included clinical examination of vocal cord status and measurement of serum calcium and parathyroid hormone levels.

## Results

The age distribution among the 66 patients who underwent thyroid surgery shows a varied spread across different age groups. The demographic pattern indicates a predominance of middle-aged and older adults among the patients. Regarding sex distribution, the data reveal a significant gender disparity. A striking 95% of the patients are female, with only 4.5% being male.

All patients underwent preoperative assessments, which confirmed normal calcium levels and vocal cord function, indicating no preexisting parathyroid disorders or vocal cord abnormalities. These baseline data are essential for postoperatively evaluating the surgical impact on these parameters. Additionally, the intraoperative use of methylene blue dye received a unanimous “Very Good" rating from surgeons, highlighting its effectiveness in precisely distinguishing anatomical structures and enhancing the accuracy and safety of thyroid surgery. Histopathological analysis revealed that 85% of the patients were diagnosed with multinodular goiter (MNG), making it the most prevalent condition. In comparison, follicular carcinoma of the thyroid (FCC) and papillary carcinoma of the thyroid (PCC) were identified in 6.1% and 9.1% of cases, respectively (Table 1).

**Table 1:** Histopathological diagnosis

Histopathological diagnosis	N (%)
Follicular cell carcinoma	4 (6.1%)
Multi nodular goiter	56 (85%)
Papillary cell carcinoma	6 (9.1%)

Postoperative outcomes for patients who underwent subtotal, near-total, or total thyroidectomy were highly favorable, with no cases of vocal cord paralysis and only 3% (two patients) experiencing transient vocal cord paresis, both recovering within a week. This suggests that methylene blue effectively minimized postoperative complications related to RLN injury. Postoperatively, all patients maintained normal serum calcium and parathyroid hormone (PTH) levels, with no Chvostek sign or carpopedal spasm, indicating stable calcium homeostasis and the absence of neuromuscular complications. Additionally, no anaphylactic reactions were reported, ensuring patient safety throughout the procedures. Regarding wound healing, 98% of patients experienced excellent outcomes, with only 1.5% (one patient) facing delayed healing, which was resolved satisfactorily (Table 2).

**Table 2:** Postoperative complication variables

Postoperative complications	N (%)
Post-operative vocal cord paralysis	0
Post-operative vocal cord paresis (recovered)	2 (3.0%)
Post-operative chovestek sign	0
Post-operative carpopedal spasm	0
Post-operative anaphylaxis	0
Post-operative wound healing	1 (1.5%)
Post-op conc fall serum calcium	0
Post-op conc fall serum parathyroid hormone	0

The statistical analysis strongly supports the efficacy of methylene blue spray in intraoperatively identifying parathyroid glands during thyroid surgery. Of the 66 specimens positive for rapid wash-out, 64 were confirmed as parathyroid tissue by histopathology, while two were fibrofatty tissue. Conversely, only one of the 66 specimens of stained blue was identified as parathyroid tissue. With a sensitivity of 98.46% and specificity of 97.01%, the spray accurately differentiates the presence or absence of parathyroid glands, as confirmed by histopathology. The PPV of 96.97% indicates a high likelihood of accurate identification when the spray signals the presence of parathyroid glands. Meanwhile, the NPV of 98.48% highlights its reliability in ruling out their presence. The overall accuracy of 97.73% reinforces methylene blue spray as a precise and effective method in thyroidectomy procedures. These findings demonstrate that methylene blue spray significantly enhances surgical precision and reduces complications, mainly when accurate identification of parathyroid glands is crucial (Tables 3-4).

**Table 3:** Methylene blue spray vs histopathology

Methylene blue spray after 5 mins	Histopathological studies	
	Positive for parathyroid gland (n = 65)	Negative for parathyroid gland (n = 67)
Rapid washout (n = 66)	64	2
Stained blue (n = 66)	1	65

**Table 4:** Diagnostic accuracy analysis of methylene blue spray

Statistic	Value	95% CI
Sensitivity	98.46%	91.72% to 99.96%
Specificity	97.01%	89.63% to 99.64%
Positive likelihood ratio	32.98	8.42 to 129.21
Negative likelihood ratio	0.02	0.00 to 0.11
Positive predictive value	96.97%	89.09% to 99.21%
Negative predictive value	98.48%	90.28% to 99.78%
Accuracy	97.73%	93.50% to 99.53%

## Discussion

The United States Food and Drug Administration has approved methylene blue. It treats several medical conditions, including acute acquired methemoglobinemia, hereditary methemoglobinemia, elderly urinary tract infection prevention, and localization of endocrine tissues and nerves. There are not many adverse effects. However, toxicity can happen if you take more than 5 mg/kg. Toxic symptoms include headaches, nausea, vomiting, disorientation, headaches, stomach discomfort, and confusion [7].

In total, 66 patients participated in the study; 64 were females (97%), and two were males (3%). The patients’ ages ranged from 20 to 65 years old, with an average age of 50.30. Every patient was free of vocal cord paresis or paralysis and had normal preoperative calcium levels. There were hardly any issues during or after surgery. Ali et al. [8] and Piromchai et al. [5] had similar results. According to Sari et al. [2], the parathyroid gland’s rich lymphovascular architecture allowed it to absorb the blue dye and return to its natural yellow hue in just three minutes. Other tissues need more time [9]. Our study yielded similar results. Piromchai et al. noted that the suspected and other areas had different blue shading. However, the surgeon was unsure about the shading in an unforeseen circumstance [5]. Therefore, we employed diluted methylene blue in our study. This made tissue differentiation easier and reduced the reactions to methylene blue. This study assessed methylene blue spray’s diagnostic usefulness in further detail. When methylene blue was sprayed, the sensitivity was 98.56%, and the specificity was 97.01%. With an accuracy of 96.73%, the PPV was 96.77%, and the NPV was 98.58%. According to our findings, methylene blue spray at the thyroid bed was very sensitive and appropriate for screening to reduce harm to the parathyroid gland and RLN. Piromchai et al. reported similar results [5].

Using anatomical landmarks [10], partial gland biopsy for pathological examination [11], optical coherence tomography during surgery [12], parathyroid-specific luminescence [13], fine needle aspiration for parathyroid analysis [14], blood parathyroid hormone levels measurement, intravenous methylene blue injection [6], photodynamic detection using 5-ALA [13], and using near-infrared fluorescence [15] are all other essential techniques used in protecting parathyroid and RLN during surgery [6]. Each alternative method offers specific benefits, such as high accuracy or low cost, but also comes with drawbacks, including potential complications, high price, or limited availability. Compared to these methods, intraoperative methylene blue spraying presents several advantages, making it a more favorable option in many cases [5]. Despite the numerous advantages highlighted by this study, anatomical identification of the parathyroid gland remains equally important. While methylene blue assists in improving the visibility and differentiation of the parathyroid gland during surgery, it cannot replace the critical importance of thorough anatomical knowledge and identification. Combining the two ensures the highest accuracy and safety in parathyroid gland identification and significantly reduces the risk of postoperative complications. 

While this study highlights numerous positive outcomes, it is essential to recognize a key limitation: the surgeons involved possess extensive experience and a profound understanding of thyroid anatomy. Their expertise would likely allow them to operate with minimal complications, regardless of methylene blue usage. As such, this study was designed as an interventional study rather than a comparative one since the surgeons’ anatomical knowledge makes it challenging to compare outcomes with and without methylene blue. Nonetheless, methylene blue undeniably aids in distinguishing anatomical structures during surgery, indicating that surgeons could significantly benefit from its application. Very few studies have explored the efficacy and diagnostic value of methylene blue spray. This research is a valuable guide, offering a blueprint for future comparative and interventional studies involving methylene blue spray.

## Conclusions

Intraoperative methylene blue spraying is a widely available, reasonably priced, and safe method for the identification and preservation of the parathyroid gland and recurrent laryngeal nerve during thyroidectomies. This technique has the potential to lower postoperative complications in total, sub-total, and near-total thyroidectomies, in addition to easing the anxiety related to thyroidectomy dissection. Its application might greatly enhance the standard of care and patient outcomes for thyroid surgery.
